# Under Chronic, High‐Dose Administration of Wenjing Decoction, the Activation of the Hepatic Nrf2/HO‐1 Pathway Mediates Increased Bilirubin Levels and Associated Reversible Neurologic Dysfunction in Mice

**DOI:** 10.1155/jt/4273765

**Published:** 2026-08-12

**Authors:** Wei Du, Min Wang, Xiaoxian Song, Ping Zhang, Xiaoli Mei, Jinping Luo, Bo Lan, Sixing Huang

**Affiliations:** ^1^ Chongqing Academy of Chinese Materia Medica, No. 34 Nanshan Road Nan’an, Chongqing 400065, China, cqacmm.com

**Keywords:** bilirubin encephalopathy, drug-induced liver injury, hyperbilirubinemia, neurotoxicity, subacute toxicity, Wenjing Decoction

## Abstract

**Background:**

Hyperbilirubinemia is a prevalent manifestation of drug‐induced liver injury, which can result in neurological complications in severe cases. Despite the extensive clinical application of Wenjing Decoction (WJD) over a millennium, there is a significant lack of systematic nonclinical safety evaluation data. Furthermore, the toxicity characteristics and mechanisms associated with long‐term high‐dose exposure remain unclear.

**Aim:**

This study seeks to conduct a systematic assessment of the toxicological characteristics of WJD via a 28‐day repeated‐dose toxicity investigation. Specifically, our focus lies in examining its impacts on hepatic and neurological functions, as well as elucidating its underlying molecular mechanisms.

**Methods:**

A bioinformatics approach was initially utilized to predict the potential molecular targets of WJD associated with hyperbilirubinemia. Subsequently, Kunming mice were randomized into either a control group or a high‐dose WJD treatment group (18.75 g/kg, equivalent to 20 times the standard human daily dose). Animals received intragastric administration of the respective treatments daily for 28 consecutive days. A subset of animals was assigned to a recovery phase to monitor the reversibility of any observed effects. Serum biochemical parameters, including total bilirubin (TBIL) and unconjugated bilirubin (UCB), hepatic oxidative stress markers such as glutathione disulfide (GSSG) and malondialdehyde (MDA), and neurobehavioral performance (evaluated via pole climbing and shuttle box tests) were systematically measured. Furthermore, Western blot, quantitative real‐time polymerase chain reaction (qRT‐PCR), and immunohistochemistry were employed to analyze the expression levels of the Nrf2/Heme oxygenase‐1 (HO‐1) signaling pathway and related inflammatory cytokines. Finally, comprehensive histopathological examinations were conducted on both liver and brain tissues.

**Results:**

Bioinformatics predictions indicate that HO‐1 is a key target. Animal experiments demonstrate that the administration of WJD results in a significant increase in serum TBIL and UCB in mice, exhibiting characteristics of nonhemolytic hyperbilirubinemia. Mild oxidative stress, characterized by increased GSSG and MDA levels, along with elevated ALT activity, occurs in the liver. This is accompanied by the activation of the Nrf2/HO‐1 pathway and the upregulation of inflammatory factors such as IL‐6, IL‐1β, and TNF‐α; however, no significant histopathological damage is observed. Regarding the nervous system, mice in the administration group show a decrease in anal temperature, impaired motor coordination, and abnormal avoidance behavior. Although the expression of HO‐1 in brain tissue is downregulated, no organic lesions are detected in the brain. All of the aforementioned abnormal indicators can be reversed during the recovery period following drug withdrawal.

**Conclusion:**

Long‐term and high‐dose exposure to WJD can induce hyperbilirubinemia in mice by activating the hepatic Nrf2/HO‐1 signaling pathway, as well as causing mild hepatic oxidative damage and neurobehavioral abnormalities. This toxic reaction is reversible, indicating that when WJD is used clinically for extended periods or at high doses, careful monitoring of bilirubin metabolism and related functional indicators is essential.

## 1. Introduction

Hyperbilirubinemia is a prevalent condition in newborns [1], typically arising from disorders of bilirubin metabolism. In adults, elevated serum bilirubin levels can result from a variety of underlying causes, including disorders of bilirubin metabolism, alcoholic liver disease, biliary stricture, biliary lithiasis, drug‐induced liver injury (DILI), hemolysis, and hepatitis [2]. If hyperbilirubinemia is not managed promptly and effectively, it can lead to rare but severe neurodevelopmental complications [3].

Wenjing Decoction (WJD) is derived from the ancient text “Synopsis of Prescriptions of the Golden Chamber: Diagnosis and Treatment of Miscellaneous Diseases in Women.” This formulation comprises *Angelica sinensis, Ligusticum chuanxiong, Paeonia lactiflora, Cinnamomum cassia, Cortex moutan, Curcuma zedoaria, Panax ginseng, Glycyrrhiza uralensis,* and *Achyranthes bidentata*. It has been utilized in human medicine for over 1000 years and was included in the “Catalogue of Ancient Classical Prescriptions” (the first batch) jointly published by the National Medical Products Administration and the National Administration of Traditional Chinese Medicine in 2018. WJD is widely employed for the treatment of primary dysmenorrhea [4] and ovulatory infertility [5]. According to the guiding principles established by the Center for Drug Evaluation of the National Medical Products Administration, safety evaluation studies must be conducted when applying for a new traditional Chinese medicine derived from an ancient classical prescription. Although WJD has been regarded as relatively safe based on several limited single‐center clinical studies with small sample sizes [6], the absence of a systematic safety evaluation necessitates the implementation of nonclinical toxicological studies.

In an unpublished nonclinical toxicological study, we observed that mice exhibited an increase in total bilirubin (TBIL) levels after receiving a constant dose of 18.75 g/kg (equivalent to 20 times the daily human dose of herbal pieces) of WJD orally for 28 consecutive days (Supporting Information S1). While WJD is known to induce hyperbilirubinemia, the extent of its effects on the liver and brain remains unclear. Consequently, this study aims to investigate the liver and brain damage associated with WJD‐induced hyperbilirubinemia and to preliminarily explore its underlying toxicity mechanisms.

## 2. Materials and Methods

### 2.1. Screening and Analysis of Potential Targets of WJD in Inducing Hyperbilirubinemia

Information on nine herbs and their protein targets was obtained from the INPUT database (https://cbcb.cdutcm.edu.cn/INPUT/) [7]. DILI is a significant cause of hyperbilirubinemia. A comprehensive list of protein targets associated with hyperbilirubinemia and DILI was compiled by searching the GeneCards database (Suite Version 5.25). High‐dose administration was employed in animal experiments. Even compounds with low bioavailability can still elicit toxic reactions due to excessive dose exposure. Consequently, all collected protein target information was preserved. The overlapping protein targets between WJD and the disease were identified using the Venn diagram tool (https://bioinfogp.cnb.csic.es/tools/venny/index.html). The identified overlapping target proteins were input into the STRING network platform Version 12.0 (https://string-db.org) with the “Homo sapiens” setting and a confidence score greater than 0.9 to predict interactions. WikiPathways and Reactome Pathways analyses were conducted using the built‐in analysis module of STRING.

### 2.2. Drugs and Reagents

WJD formulation was provided by Chongqing Attractiveness & Distinctiveness Health Technology Co., Ltd. (Chongqing, China). Reference standards were supplied by the National Institutes for Food and Drug Control (Beijing, China). Hematology analysis reagents were sourced from Sysmex Corporation (Kobe, Japan), while biochemical assay kits were obtained from Beckman Coulter Laboratory Systems (Suzhou, China) Co., Ltd. Nissl staining reagents were purchased from Beijing Biosynthesis Biotechnology Co., Ltd., and primers were synthesized by Sangon Biotech (Shanghai, China) Co., Ltd. Primary and secondary antibodies were provided by Proteintech Group, Inc.

### 2.3. Animals

Kunming mice were purchased from Beijing Vital River Laboratory Animal Technology Co., Ltd. (Beijing, China). Mice were reared in a controlled environment with constant temperature (20∼26°C), constant humidity (30∼70%), alternating light and dark (12 h/12 h), and standard pellet feed and drinking water. All animal use was carried out in accordance with the animal guidelines and approved by the Animal Ethics Committee of Chongqing Academy of Chinese Materia Medica.

### 2.4. Repeated Administration in Mice and Experimental Design

The doses for the toxicological study were prepared prior to administration. The WJD powder was dissolved in purified water to achieve a final solution concentration of 625 mg/mL. In accordance with the quality control requirements set by the WJD manufacturer, the content of glycyrrhizic acid in the prepared WJD was quantified using high‐performance liquid chromatography (HPLC). The acceptable content range was determined to be between 1896.7 and 2566.1 mg. Specifically, a Shimadzu LC‐40BX3 high‐performance liquid chromatograph (Japan) was utilized. The chromatographic column employed was a Shim‐pack GIST C18 (2.1 × 100 mm, 2 μm). The mobile phase consisted of acetonitrile and 0.1% phosphoric acid, utilizing gradient elution (Supporting Information S2). The detection wavelength was set at 250 nm, with a flow rate of 0.42 mL/min and a column temperature maintained at 30°C. The injection volume was 1.5 μL.

The mice were divided into two groups at random, with eight mice of each sex in each group. Four mice were sacrificed at the conclusion of the administration period (Day 29 [D29]), while the remaining four were sacrificed after the recovery period (Day 57 [D57]). Over the course of 28 days, the animals in the WJD group received a constant dose of 18.75 g/kg, which is equivalent to 20 times the daily human dose of the decoction pieces. The control group was gavaged with an equal volume of purified water. Given the unclear mechanism of WJD‐induced injury and the aim to reduce the number of animals used, a positive control group was not established.

### 2.5. Measurement of Rectal Temperature in Mice

Mice were gently restrained, and a lubricated thermometer probe was inserted into the rectum to a depth of approximately 2 cm. The temperature was recorded after 3 min to establish the rectal temperature.

### 2.6. Pole Climbing Test

The pole climbing test was employed to assess the limb coordination abilities of mice in each group [8]. The animals were positioned on a smooth, vertically oriented metal pole (with a diameter of 0.80 cm and a length of 65.0 cm), facing downward, and were permitted to descend naturally. The motor performance of the mice was then evaluated and scored. Following three adaptation trials, the scores from the 4th and 5th attempts were recorded, and the average score was included in the statistical analysis. The scoring criteria are presented in Table 1.

**TABLE 1:** Pole test.

Score	Standard
0	Step by step, down the pole
1	Slide down the pole
2	Cannot get a grip on the pole
3	Loss of the righting reflex

### 2.7. Shuttle Box Test

The trained mice were placed in a shuttle box, which is designed with areas for light and sound stimulation, electrical stimulation, and safety. An escape to the safety area during light and sound stimulation is classified as active avoidance behavior, whereas an escape following electrical stimulation is classified as passive avoidance behavior. The Shuttle Box Test Video Analysis System (Shanghai Jiliang Software Science & Technology Co., Ltd., China) was employed to analyze the active and passive avoidance behaviors of the mice over a duration of 5 min.

### 2.8. Hematology

During the blood collection process in mice, approximately 0.5 mL of blood was obtained from the orbital sinus using a blood collection needle (D29). The collected blood was stored in heparinized EDTA tubes for the measurement of hematological parameters, which were analyzed using the XT‐2000i fully automatic animal blood analyzer (Japan). The parameters measured included the total red blood cell (RBC) count, hemoglobin (HGB), and reticulocyte count (RET#). RBC counts were measured at D29 specifically to determine whether the elevated serum bilirubin was hemolytic in origin. Once nonhemolytic hyperbilirubinemia was confirmed at this time point, RBC assessment was not repeated at D57, as additional measurement would have yielded no further benefit beyond increasing workload.

### 2.9. Serum Biochemistry

Approximately 0.5 mL of blood was extracted from each animal and centrifuged at 3000 r/min for 15 min to obtain the serum. TBIL, direct bilirubin (DBIL), aspartate aminotransferase (AST), alanine aminotransferase (ALT), and alkaline phosphatase (ALP) levels in the collected serum were analyzed using an AU480 automatic biochemical analyzer (USA). The unconjugated bilirubin (UCB) was subsequently calculated.

### 2.10. Gross Assessment and Histopathology

All animals underwent a comprehensive gross necropsy. This procedure included a meticulous examination of the external body surface; all natural orifices; and the cranial, thoracic, and abdominal cavities with their respective contents. Following the necropsy, the liver and brain were excised and cleared of adherent tissues. Subsequently, target organs were fixed in 4% paraformaldehyde for histological processing. Fixed tissues were embedded in paraffin and sectioned using a Leica RM2235 rotary microtome. The resulting sections were stained with hematoxylin and eosin (HE) for general histopathology; brain sections were additionally subjected to Nissl staining to assess neuronal morphology. Finally, stained slides were evaluated under light microscopy using a Mias‐2000 pathological image analysis system.

### 2.11. Immunohistochemical Staining Procedure

Liver tissue sections were incubated with primary Nrf2 antibody (dilution 1:1000; Servicebio, China) overnight at 4°C. Following three washes with PBS, the sections were incubated with secondary antibody (dilution 1:200) at room temperature for 50 min. Immunoreactivity was visualized using a DAB detection kit, followed by counterstaining with hematoxylin. The sections were subsequently dehydrated through an ethanol gradient, cleared in xylene, and mounted. Stained slides were examined under a microscope.

### 2.12. Parameters of Oxidative Balance Markers in Liver Tissue

Reduced glutathione (GSH) functions as a key cellular antioxidant, whereas oxidized GSH (glutathione disulfide [GSSG]) and malondialdehyde (MDA) serve as established biomarkers of oxidative stress [9]. Hepatic levels of GSH, GSSG, and MDA were quantified using commercial assay kits (Beyotime Biotechnology, China) according to the manufacturer’s instructions.

### 2.13. RNA Extraction From Liver and Quantitative Real‐Time Polymerase Chain Reaction (qRT‐PCR)

Total RNA was extracted from homogenized mouse liver tissues using TRIzol reagent according to the manufacturer’s protocol. The RNA was subsequently reverse‐transcribed into cDNA for qRT‐PCR analysis.

Amplification was performed using the following thermal cycling conditions: initial predenaturation at 95°C for 30 s, followed by 45 cycles of denaturation at 95°C for 10 s, annealing at 60°C for 30 s, and extension at 72°C for 30 s. Primer specificity was confirmed by melt curve analysis. The threshold cycle (Ct) values were recorded, and each sample was run in triplicate to calculate the mean value. Relative gene expression levels were determined using the 2^ΔΔCt^ method. The specific primer sequences are listed in Supporting Information S3.

### 2.14. Western Blotting (WB) Assay

Total protein was extracted from mouse liver and brain tissues by lysis in RIPA buffer. Protein concentration was determined using a BCA assay. Subsequently, samples were mixed with 5 × SDS‐PAGE loading buffer and denatured by heating at 95°C for 5 min. Equal amounts of protein were resolved by SDS‐PAGE and transferred onto PVDF membranes. Membranes were blocked with 5% (w/v) nonfat milk and incubated with primary and secondary antibodies. Protein bands were visualized using an ECL chemiluminescence detection kit.

### 2.15. Statistical Analysis

All data were analyzed using R programming. Descriptive statistics were used to submit the data for normality testing. Normality was assessed by the Shapiro–Wilk test and homogeneity of variances by Levene’s test for each dataset. An independent samples *t*‐test was performed on the toxicity data that conformed to the normal distribution. For the toxicity data that did not conform to the normal distribution, nonparametric Kruskal–Wallis test, Mann–Whitney *U* test, and Wilcoxon rank‐sum test (when appropriate) were used to compare the means of each row in the repeated experiments. Data were expressed as mean ± standard error of the mean (S.E.M). A probability level of *p* < 0.05 was considered significant.

## 3. Results

### 3.1. Predictions Indicate That WJD‐Induced Hyperbilirubinemia is Regulated via a Multitarget Mechanism, Identifying Heme Oxygenase as a Key Protein for Experimental Validation

**FIGURE 1:**
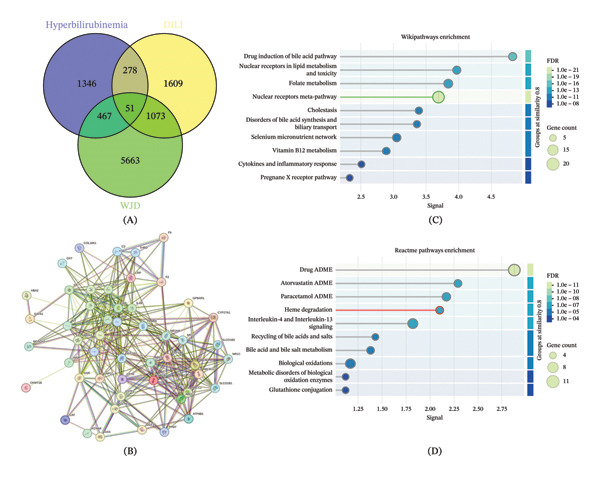
Bioinformatics prediction results of WJD‐induced hyperbilirubinemia. (A) Venn diagram of common targets among WJD, hyperbilirubinemia, and DILI. (B) Protein–protein interaction network of potential targets for WJD‐induced hyperbilirubinemia. (C) Top 10 signaling pathways enriched by potential targets (WikiPathways analysis). (D) Top 10 signaling pathways enriched by potential targets (Reactome Pathways analysis).

The nine herbal components were found to act on 7277 nonredundant protein targets. The GeneCards database identified 2142 proteins associated with hyperbilirubinemia and 3011 proteins associated with DILI. A total of 51 proteins were identified as common targets intersecting WJD, hyperbilirubinemia, and DILI (Figure 1A). The biological activities of these 51 proteins are highly likely to represent the targets through which WJD induces hyperbilirubinemia. However, the potential interactions and biological functions among these targets required further prediction.

STRING platform analysis predicted that the 51 potential targets for WJD‐induced hyperbilirubinemia are interconnected, forming a complex network (Figure 1B). WikiPathways analysis highlighted the top 10 signaling pathways (Figure 1C). The targets were most significantly enriched in the “Nuclear receptors meta‐pathway,” which includes heme oxygenase (HMOX1 or Heme oxygenase‐1 [HO‐1]), GSR, IFNG, SLCO1B1, IL1B, ABCB4, GSTM1, NR1I2, NR1I3, THBD, G6PD, GSTP1, PPARA, TNF, SLC2A1, NR1H4, ABCB1, ABCC4, ABCC2, ABCB11, and CYP3A4. Reactome Pathways analysis also displayed the top 10 signaling pathways (Figure 1D). Among these, aberrant regulation of “Heme degradation” was directly associated with elevated bilirubin levels, involving targets such as HO‐1, SLCO1B1, SLCO1B3, ABCC2, and ABCG2. As HO‐1 serves as a core target in both the nuclear receptors meta‐pathway and heme degradation, it was selected as the focal protein for experimental validation.

### 3.2. The Prepared WJD was Subjected to Rigorous Quality Control

**FIGURE 2:**
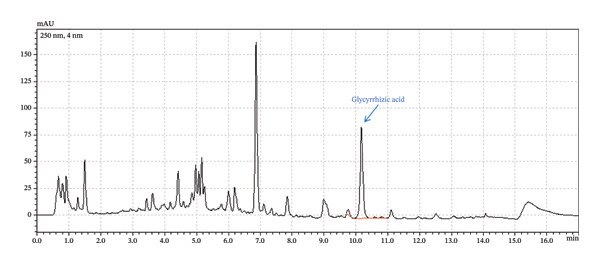
Retention time of the glycyrrhizic acid chromatographic peak.

The retention time of the glycyrrhizic acid chromatographic peak was 10.180 min (Figure 2), which fell within the timeframe stipulated by the Chinese Pharmacopoeia (2025 Edition). The concentration of glycyrrhizic acid in the 625 mg/mL WJD solution was determined to be 2403.9 μg/mL, satisfying the required content specifications.

### 3.3. WJD Induces Nonhemolytic Hyperbilirubinemia

**FIGURE 3:**
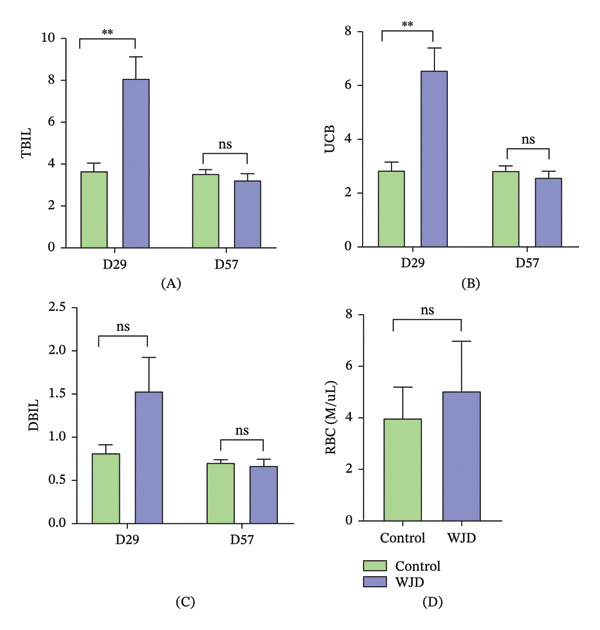
Changes in bilirubin and red blood cells. (A) TBIL (D29 and D57). (B) UCB (D29 and D57). (C) DBIL (D29 and D57). (D) RBC (D29). Data are expressed as mean ± standard error of the mean (S.E.M.) (*n* = 8 animals/group). ^∗∗^ *p* < 0.01 compared to the control group at the same time point.

Mice were continuously exposed to WJD for 28 days. Both TBIL and UCB levels were significantly elevated compared to the control group (*p* < 0.01) (Figures 3A and B). Although DBIL showed an upward trend, the change was not statistically significant (*p* > 0.05) (Figure 3C). All parameters returned to baseline naturally by D57. RBC counts, an indicator of hemolysis, showed no significant difference between the two groups at the end of the exposure period. RBC levels were not assessed at the end of the natural recovery period.

### 3.4. WJD Causes Mild, Reversible Oxidative Liver Injury

**FIGURE 4:**
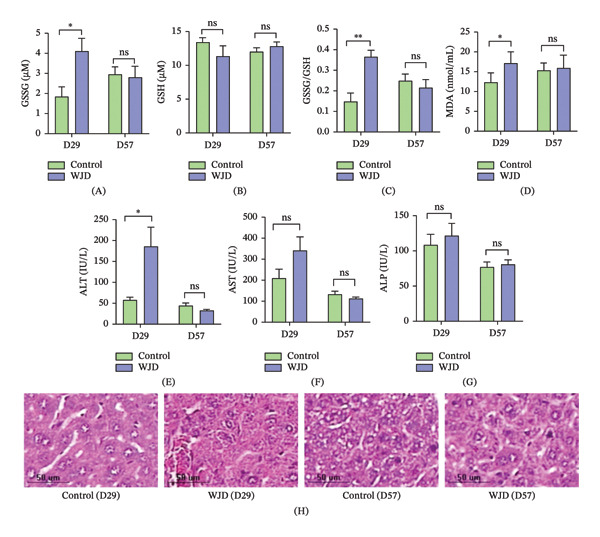
Hepatic oxidative stress, biochemical markers of liver injury, and histopathological changes. (A) GSSG. (B) GSH. (C) GSSG/GSH. (D) MDA. (E) ALT. (F) AST. (G) ALP. (H) HE staining of liver tissues (× 100). Data are expressed as mean ± standard error of the mean (S.E.M.) (*n* = 8 animals/group). ^∗^ *p* < 0.05 and ^∗∗^ *p* < 0.01 compared to the control group at the same time point. Scale bar: 50 μm.

After 28 days of exposure, the levels of oxidative stress markers—GSSG, GSSG/GSH ratio, and MDA—were significantly elevated in WJD‐treated mice (*p* < 0.05, *p* < 0.01) (Figures 4A, B, and D). Although GSH levels decreased, the change was not statistically significant (*p* > 0.05) (Figure 4C). These stress responses were reversible. Regarding liver injury markers, ALT levels were significantly elevated at the end of the dosing period (*p* < 0.05) (Figure 4E). AST and ALP showed only an upward trend without statistical significance (*p* > 0.05) (Figures 4F and G). By D57, the expression levels of ALT, AST, and ALP were comparable between the two groups. HE staining and microscopic observation (× 100) revealed no obvious liver injury in either group at the end of the dosing period or at the end of the recovery period.

### 3.5. The Nrf2/HO‐1 Signaling Pathway Mediates Hepatic Oxidative Injury

**FIGURE 5:**
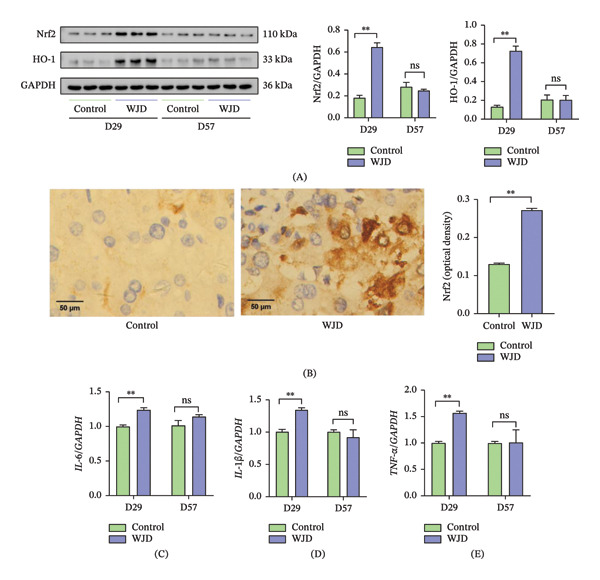
Alterations in the Nrf2/HO‐1 signaling pathway in liver tissue. (A) Western blot analysis of Nrf2 and HO‐1. (B) Immunohistochemical staining results of Nrf2 around liver tissue bile ducts (× 400). (C–E) Quantitative real‐time PCR analysis of IL‐6, IL‐1*β*, and TNF‐*α*. Data are expressed as mean ± standard error of the mean (S.E.M.) (*n* = 3 animals/group). ^∗∗^ *p* < 0.01 compared to the control group at the same time point. Scale bar: 50 μm.

Compared to the control group, the expression levels of Nrf2 and HO‐1 in the livers of WJD‐treated mice were significantly upregulated at D29 (*p* < 0.01) (Figure 5A). Microscopic observation (× 400) revealed a significant increase in Nrf2 immunopositivity around the bile ducts, characterized by a marked accumulation of brownish‐yellow granules surrounding the nuclei (*p* < 0.01) (Figure 5B). A‐In addition, the expression levels of IL‐6, IL‐1*β*, and TNF‐*α* were upregulated (*p* < 0.01) (Figure 5C–E). At the end of the recovery period, the expression levels of indicators related to the Nrf2/HO‐1 signaling pathway were comparable between the two groups, with no significant difference observed.

### 3.6. WJD‐Induced Hyperbilirubinemia Leads to Reversible Neurobehavioral Alterations in Mice

**FIGURE 6:**
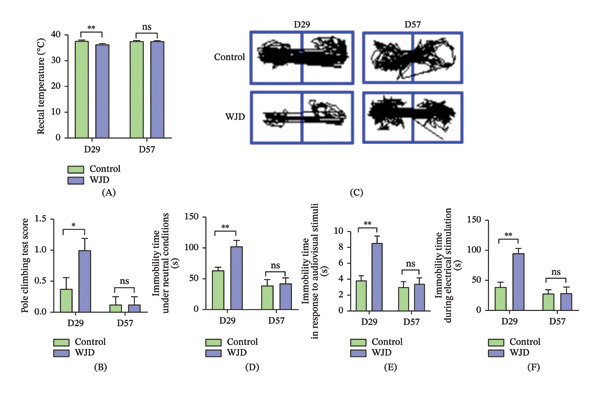
Rectal temperature and behavioral changes in mice. (A) Rectal temperature. (B) Pole climbing test score. (C) Mouse movement trajectories. (D) Immobility time under neutral conditions. (E) Immobility time in response to audiovisual stimuli. (F) Immobility time during electrical stimulation. Data are expressed as mean ± standard error of the mean (S.E.M.) (*n* = 8 animals/group). ^∗∗^ *p* < 0.01 compared to the control group at the same time point.

Compared to the control group, the rectal temperature of WJD‐treated mice was significantly reduced (*p* < 0.01) (Figure 6A). Following continuous WJD exposure, the pole climbing test scores were significantly elevated (*p* < 0.05) (Figure 6B). Concurrently, the mice exhibited impaired active and passive avoidance capabilities, manifested as a significant increase in immobility time under neutral conditions, immobility time in response to audiovisual stimuli, and immobility time during electrical stimulation (*p* < 0.01) (Figures 6C–F). These behavioral abnormalities were reversible, returning to baseline levels.

### 3.7. HO‐1 Downregulation in the Brain Does Not Lead to Histopathological Injury

**FIGURE 7:**
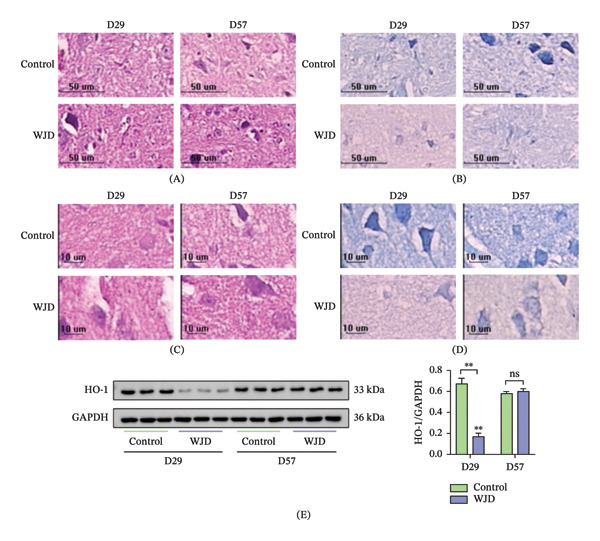
Histopathological observation and HO‐1 changes in mouse brain tissue. (A) Mouse brainstem (H&E staining, × 100). (B) Mouse brainstem (Nissl staining, × 100). (C) Mouse cerebral cortex (H&E staining, × 200). (D) Mouse cerebral cortex (Nissl staining, × 200). (E) Western blot analysis of HO‐1. Data are expressed as mean ± standard error of the mean (S.E.M.) (*n* = 3 animals/group). ^∗∗^ *p* < 0.01 compared to the control group at the same time point.

HE staining and Nissl staining revealed no obvious injury in the mouse brainstem or cortex (Figures 7A–D). However, compared to the control group, the expression level of HO‐1 in WJD‐treated mice was significantly downregulated at the end of the dosing period (*p* < 0.01) (Figure 7E), which was reversible.

## 4. Discussion

Bilirubin is a yellow–orange bile pigment and a catabolic product of heme metabolism, traditionally regarded as a waste product and a marker of liver injury [10]. Approximately 85% of heme is derived from the degradation of HGB in RBCs, while the remainder originates from ineffective erythropoiesis and the breakdown of other hemoproteins such as cytochromes, myoglobin, and catalase [11]. The conversion of heme to bilirubin is a two‐step reaction: first, microsomal HO‐1 in the reticuloendothelial system converts heme to biliverdin, which is subsequently reduced to UCB by the enzyme biliverdin reductase [12]. UCB is lipophilic and is transported to the liver tightly bound to albumin [13]. In hepatocytes, UCB dissociates from albumin and binds to proteins of the GSH‐S‐transferase (GST) family, which present it for conjugation, preventing its efflux from the liver. Subsequently, UCB is conjugated with one or two glucuronic acid molecules by UDP‐glucuronosyltransferase (UGT1A1), forming bilirubin monoglucuronide and bilirubin diglucuronide, respectively [14]. Conjugation increases the solubility of bilirubin in plasma, thereby enhancing its excretion. Conjugated bilirubin (CB) is then pumped into bile via an energy‐dependent process requiring Multidrug resistance–associated Protein 2 (MRP2) [15]. This process also reduces the ability of bilirubin to diffuse across the blood–brain barrier [16]. Consequently, dysfunction in any of these conjugation steps can lead to hyperbilirubinemia. Specifically, one or a combination of three main pathophysiological conditions—increased bilirubin production, impaired bilirubin uptake, or impaired bilirubin conjugation—can result in unconjugated hyperbilirubinemia.

The present study demonstrates that well‐quality‐controlled WJD induces hyperbilirubinemia in mice. WJD‐induced hyperbilirubinemia is characterized by significantly elevated TBIL and UCB levels (Figures 3A and B), with no obvious erythrocyte injury (Figure 3D). We further examined other indicators of liver function; the significant elevation of ALT (Figure 4E) and the upward trends in AST and ALP (Figures 4F and G) suggest injury related to hepatic biliary function [17]. Histopathological examination revealed no obvious organic injury (Figure 4H), indicating that the impairment of biliary function is mild.

HO‐1 protein is present not only in microsomes but also in hepatic mitochondria [18], where it prevents heme accumulation and toxicity [19]. HO‐1 expression can be induced by various cellular events, including stress [20]. Upon stimulation, Nrf2 replaces BACH1 at binding sites on the HO‐1 promoter, leading to increased HO‐1 gene expression [21]. When ROS are excessive, GSH is oxidized to GSSG, leading to elevated GSSG levels; if GSSG is not timely reduced back to GSH, it can lead to protein disulfide bond formation via “thiol stress,” disrupting cellular structure and function [22]. The oxidative parameters GSSG, MDA, and the GSSG/GSH ratio were significantly elevated in the liver tissues of mice at the end of WJD treatment (Figures 4A–D), indicating that hepatocytes are undergoing oxidative stress. Immunohistochemical observation showed increased Nrf2 expression around the nucleus (Figure 5B), a prerequisite for the initiation of the Nrf2/HO‐1 signaling pathway; the activation of this pathway exacerbates cholestatic liver injury [23]. Furthermore, Western blot analysis revealed upregulated expression levels of Nrf2 and HO‐1 (Figure 5A) and downstream inflammatory factors (Figures 5C–E), suggesting that HO‐1 overexpression is a significant mechanism in WJD‐induced hyperbilirubinemia, and that Nrf2/HO‐1 pathway activation is a mechanism of WJD‐induced liver injury. In addition, GST is a multifunctional dimeric protein family that catalyzes the covalent binding of GSH to electrophilic compounds, generating more hydrophilic, excretable conjugates [24]. The trend of decreased GSH may also lead to impaired bilirubin conjugation.

Mechanistically, the elevated proinflammatory cytokines IL‐6, IL‐1β, and TNF‐α observed here can further reinforce oxidative stress and Nrf2/HO‐1 pathway activation. These cytokines are known to stimulate NADPH oxidases and mitochondrial ROS production, thereby exacerbating the oxidative milieu that drives Nrf2 nuclear translocation [25]. Reciprocally, while Nrf2 is generally cytoprotective, its sustained activation under pathological conditions can upregulate HO‐1 beyond physiological needs. Excessive HO‐1 activity liberates free iron and carbon monoxide, which can promote prooxidant reactions and mitochondrial dysfunction, respectively [26]. This creates a vicious cycle wherein oxidative stress and inflammation perpetuate one another. Moreover, the interplay between Nrf2 and NF‐κB—the master regulator of IL‐6, IL‐1β, and TNF‐α—is critical. Under unresolved oxidative stress, Nrf2 can lose its ability to suppress NF‐κB, and the two pathways may instead co‐activate, leading to simultaneous upregulation of HO‐1 and inflammatory mediators [27]. In the context of hyperbilirubinemia, HO‐1 overexpression directly increases bilirubin production via heme degradation [28]. When this occurs alongside compromised bilirubin conjugation and excretion—as suggested by the decreased GSH and elevated GSSG levels in this study—the net result is an accumulation of bilirubin in the bloodstream. Thus, the Nrf2/HO‐1 pathway activation does not merely accompany, but actively contributes to, the inflammatory and hyperbilirubinemic phenotype induced by WJD, establishing a feed‐forward loop of hepatocyte injury, inflammation, and impaired bile pigment clearance.

Hearing impairment, cognitive deficits, and loss of voluntary movement and communication, known as kernicterus spectrum disorder, represent severe and permanent injuries to the central nervous system from exposure to high levels of bilirubin [29]. UCB can cross the blood–brain barrier [30], leading to bilirubin exposure in the central nervous system. The cortex and brainstem are common sites of bilirubin injury [31]. Recent studies indicate that the brainstem is particularly sensitive to bilirubin [32]. Therefore, this study focused on injury to the brainstem and cortex. At the end of WJD exposure, mice exhibited coordination abnormalities, increased immobility time under neutral conditions, increased immobility time in response to audiovisual stimuli, and increased immobility time during electrical stimulation (Figures 6B–F), indicating the neurotoxicity of WJD‐induced hyperbilirubinemia. Histopathological examination (Figures 7A–D) revealed no obvious neuronal injury in the brainstem and cortex, and given the reversibility of the behavioral changes, the neurotoxicity is considered mild.

Under physiological conditions, HO‐1 expression is limited to peripheral glial cells and neurons [33] and serves as an important neuroprotective defense mechanism [34]. HO‐1 defense profiles include both high and low expression [35], with low expression serving to mitigate oxidative damage. It is well known that brain function is highly dependent on oxygen [36]; HO‐1 degrades heme‐derived carbon monoxide and iron, which may lead to cerebral hypoxia [37]. Thermoregulation is typically associated with hypothalamic balance, and hypoxia is a significant factor inducing regulatory imbalance [38]. Rectal temperature measurements revealed a decrease in body temperature in mice at the end of WJD exposure (Figure 6A). We hypothesize that high‐dose WJD exposure induced cerebral hypoxia, leading to substantial consumption of HO‐1 in the brain, which explains the observed low expression of HO‐1 protein in brain tissue (Figure 7E). This also appears to explain the presence of behavioral abnormalities (Figures 6B–6F) in the absence of organic brain tissue injury (Figures 7A–7D). However, we acknowledge that this hypothesis regarding cerebral hypoxia is speculative, as specific hypoxia‐related indicators (e.g., HIF‐1α and lactate) were not measured in this study. Future investigations incorporating these markers are necessary to validate this mechanism.

This study focused on HO‐1, and combined with the positive findings mentioned above, it indicates that the prediction of HO‐1 as a protein of interest for experimental validation (Figure 1) was proactive and fruitful.

As noted in the Introduction, this work represents two follow‐up studies built upon previously unpublished preliminary research. Using distinct batches of the WJD test article, we observed consistent toxicological profiles across identical dose levels, which demonstrates robust interbatch reproducibility. These findings are intended to alert clinical practitioners to the contraindications associated with WJD administration, particularly the need to monitor potential impacts on bilirubin metabolism and the function of related organs during prolonged or high‐dose treatment. Future research will prioritize investigations into the material basis underlying WJD’s toxic effects.

## 5. Conclusion

This study demonstrates that long‐term, high‐dose WJD (18.75 g/kg, equivalent to 20 times the human daily dose) induces nonhemolytic hyperbilirubinemia in mice, characterized by significantly elevated serum TBIL and UCB levels. The underlying mechanism is associated with the activation of the hepatic Nrf2/HO‐1 pathway, increased bilirubin production, and impaired bilirubin conjugation. Concurrently, mice exhibited reversible neurobehavioral abnormalities, including decreased body temperature, reduced motor coordination, and impaired avoidance behavior, in the absence of organic injury. Given that these toxic effects are reversible, clinical application of WJD, particularly during long‐term or high‐dose administration, warrants monitoring of potential effects on bilirubin metabolism and related organ function.

**Nomenclature**
ALP: Alkaline phosphatase
ALT: Alanine aminotransferase
AST: Aspartate aminotransferase
DBIL: Direct bilirubin
DILI: Drug‐induced liver injury
GSH: Reduced glutathione
GSSG: Glutathione disulfide
HE: Hematoxylin and eosin
HGB: Hemoglobin
HO‐1/HMOX1: Heme oxygenase 1
IL: Interleukin
MDA: Malondialdehyde
Nrf2: Nuclear factor erythroid 2–related factor 2
qRT‐PCR: Quantitative real‐time PCR
RBC: Red blood cell
RET#: Reticulocyte count
TBIL: Total bilirubin
TNF‐α: Tumor necrosis factor‐alpha
UCB: Unconjugated bilirubin
WJD: Wenjing Decoction

## Author Contributions

Wei Du: writing–original draft, project administration, methodology, and data curation. Min Wang: writing–original draft, project administration, methodology, and data curation. Xiaoxian Song, Ping Zhang, Xiaoli Mei, and Jinping Luo: project administration and methodology. Bo Lan: writing–review and editing, supervision, investigation, data curation, and conceptualization. Sixing Huang: writing–review and editing, supervision, investigation, funding acquisition, data curation, and conceptualization.

## Funding

This research received no specific grant from any funding agency in the public, commercial, or not‐for‐profit sectors.

## Ethics Statement

This study has been reviewed and formally approved by the Ethics Committee for Experimental Animals at the Chongqing Academy of Chinese Materia Medica (Approval No. YLS2024‐01). The entire research process strictly adhered to the ethical principles outlined in the Regulations on the Administration of Experimental Animals of the People’s Republic of China, as well as the internal ethical guidelines and operational standards of the Chongqing Academy of Chinese Materia Medica. Throughout the study, all procedures involving experimental animals were implemented under the direct supervision of the aforementioned Ethics Committee to ensure full adherence to institutional ethical requirements, animal welfare protection, and the scientificity and rigor of experimental operations.

## Consent

The authors have nothing to report.

## Conflicts of Interest

The authors declare no conflicts of interest.

## Supporting Information

Additional supporting information can be found online in the Supporting Information section.

## Supporting information


**Supporting Information** Table S1: Preliminary experimental results of repeated gavage administration of WJD. Table S2: Gradient elution table. Table S3: The primers used.

## Data Availability

The data that support the findings of this study are available from the corresponding author upon reasonable request.
