# Supplementary material for: Under Chronic, High‐Dose Administration of Wenjing Decoction, the Activation of the Hepatic Nrf2/HO‐1 Pathway Mediates Increased Bilirubin Levels and Associated Reversible Neurologic Dysfunction in Mice
Source: J Toxicol. 2026 Aug 12;2026:4273765. doi: 10.1155/jt/4273765 (PMC13469760; doi:10.1155/jt/4273765)
Supplement: Supplementary file 1 [file JT-2026-4273765-s001.doc]

**Supplementary Table**

S1 Preliminary experimental results of repeated gavage administration of Wenjing Decoction (WJD)

| Group | *n* | Test substance | Dose | TBIL |
| --- | --- | --- | --- | --- |
| Control | 6 | Purified water | / | 2.09±0.86 |
| Low-dose | 6 | WJD | 9.37g/kg | 2.14±0.61 |
| medium-dose | 6 | WJD | 18.75g/kg | 6.02±0.73** |
| High-dose | 6 | WJD | 37.5g/kg | 6.21±0.73** |

Note: The table highlights a statistically significant elevation in Total Bilirubin (TBIL) levels in the treatment group compared to the control (*p <* 0.01), consistent with the hyperbilirubinemia phenotype described in the main text.

S2 Gradient elution table

| Time (min) | Acetonitrile (%) | 0.1% Phosphoric Acid (%) |
| --- | --- | --- |
| 0.0~5.0 | 7→34 | 93→66 |
| 5.0~12.0 | 34→43 | 66→57 |
| 12.0~12.5 | 43→80 | 57→20 |
| 12.5~13.5 | 80 | 20 |
| 13.5~14.0 | 80→7 | 20→93 |
| 14.0~17.0 | 7 | 93 |

Note: This table details the gradient elution program employed to ensure accurate quality control of the test article prior to administration.

S3 The primers used

| Primers | Sequences |
| --- | --- |
| R-IL-1β-F | 5'-GCCACCTTTTGACAGTGATGAG-3' |
| R-IL-1β-R | 5'-GACAGCCCAGGTCAAAGGTT-3' |
| R-IL-6-F | 5'-AGTTCCTCTCTGCAAGAGACTTC-3' |
| R-IL-6-R | 5'-AAGTAGGGAAGGCCGTGGTT-3' |
| R-TNF-α-F | 5'-TAGCCCACGTCGTAGCAAAC-3' |
| R-TNF-α-R | 5'-ACAAGGTACAACCCATCGGC-3' |

Note: Sequences of primers used for quantitative real-time PCR (qRT-PCR) analysis of gene expression in mouse liver tissues.
